# Dengue and Zika Viruses: Epidemiological History, Potential Therapies, and Promising Vaccines

**DOI:** 10.3390/tropicalmed5040150

**Published:** 2020-09-23

**Authors:** Nelly M. Silva, Nuno C. Santos, Ivo C. Martins

**Affiliations:** Instituto de Medicina Molecular, Faculdade de Medicina, Universidade de Lisboa, Av. Prof. Egas Moniz, 1649-028 Lisbon, Portugal; imm@medicina.ulisboa.pt

**Keywords:** dengue, Zika, *Flavivirus*, epidemiology, *Aedes*

## Abstract

Dengue virus (DENV), which can lead to fatal hemorrhagic fever, affects 390 million people worldwide. The closely related Zika virus (ZIKV) causes microcephaly in newborns and Guillain-Barré syndrome in adults. Both viruses are mostly transmitted by *Aedes albopictus* and *Aedes aegypti* mosquitoes, which, due to globalization of trade and travel alongside climate change, are spreading worldwide, paving the way to DENV and ZIKV transmission and the occurrence of new epidemics. Local outbreaks have already occurred in temperate climates, even in Europe. As there are no specific treatments, these viruses are an international public health concern. Here, we analyze and discuss DENV and ZIKV outbreaks history, clinical and pathogenesis features, and modes of transmission, supplementing with information on advances on potential therapies and restraining measures. Taking advantage of the knowledge of the structure and biological function of the capsid (C) protein, a relatively conserved protein among flaviviruses, within a genus that includes DENV and ZIKV, we designed and patented a new drug lead, pep14-23 (WO2008/028939A1). It was demonstrated that it inhibits the interaction of DENV C protein with the host lipid system, a process essential for viral replication. Such an approach can be used to develop new therapies for related viruses, such as ZIKV.

## 1. Introduction

Vector-borne diseases cause more than 700,000 deaths per year. They contribute to more than 17% of all infectious diseases, especially affecting the poorest populations [1]. Outbreaks of dengue, Zika, and other related infectious diseases have been occurring in tropical and subtropical regions and are now expanding to other areas [1]. Importantly, dengue and Zika viral infections not only have an impact on people lives, causing hospitalizations and deaths, but also on the society and economy, temporarily stagnating it to some extent, not only due to the direct deaths, but also due to the long period required for victims to fully recover. As tropical regions are mostly comprised of developing countries and as no specific treatments are available, the impact of dengue and Zika outbreaks on the socio-economic sector is disproportionate, strongly affecting already vulnerable societies less able to cope with the added burden [2,3,4,5]. One study summed up dengue costs of 18 countries and concluded that, in 2015, they totaled US$ 3.3 billion (purchasing power parity) [5]. To compare, the World Bank estimated that the short-term economic impact of Zika epidemics in 2016 in Latin American and Caribbean region was US$ 3.5 billion [4]. There is, therefore, an urgent need to find effective treatments and/or prophylactic measures that are available for all people to attenuate dengue and Zika infections’ burden on health systems and finances of affected countries and populations.

*Aedes* spp. mosquitoes, specially *Aedes albopictus* and *Aedes aegypti*, the main vectors of dengue virus (DENV) and Zika virus (ZIKV) transmission, are expanding worldwide [2,6,7,8,9,10,11]. These vectors are no longer confined to tropical and subtropical regions, making them and the diseases they carry commonly recognized [10,11]. DENV and ZIKV infections are therefore of concern for national health services on most continents, as epidemic outbreaks can cause major health impacts and have enormous costs [2,3,4,5].

## 2. Epidemiology of Dengue and Zika

The likelihood of outbreaks of arthropod-borne viruses (such as DENV and ZIKV) to occur is directly related to the abundance of vectors for transmission and the availability of non-immune people. Increased urbanization, movement of people and goods, environmental changes, and biological challenges (e.g., mosquito resistance to insecticides) are fueling the spread of these viral vectors to new regions, as discussed below. A historical perspective of the presence of the disease worldwide and the expansion of these vectors is also presented, showing that, for example, dengue has been a problem in Europe in the past. It is likely to become a major issue in the coming decades, given the mosquito presence and DENV spread, as analyzed below.

### 2.1. Dengue History and Epidemiology Worldwide

The history of dengue is unclear and complicated. Part of the problem is that some of its clinical features are similar to other febrile viral illnesses, including Chikungunya, an infection caused by an arbovirus transmitted by the same vector. The earliest clinical reports compatible with dengue fever were first published during the Jin Dynasty (265 to 420 A.D.) [12]. The illness was named “water poison” by the Chinese population, probably due to an association with flying insects and water [12,13]. Later on, suspected dengue-like epidemics were reported in 1635 and 1699 in the French West Indies and Panama, respectively. The first confirmed epidemics of dengue fever occurred in 1779-1780, spanning three continents: Africa, Asia, and North America [6]. The almost simultaneous occurrence of these outbreaks indicates that the distribution of the virus and its vector on the tropics is not recent. Only in 1828, during an epidemic in Cuba, the name dengue was coined to this disease (initially it was named Dunga). However, the name was probably originated earlier in Spain, as stated in a letter by the Queen of Spain, María Luisa de Parma, in 1801, stating that she may be suffering from it [14,15]. Between that date and the first isolation of dengue viruses in Japan, in 1943 and, in Hawaii, in 1945, there were several outbreaks that were clinically consistent with dengue. [16,17]. Until 1940, major epidemics generally occurred with long intervals (10 to 40 years) between them, perhaps due to the slow transport of virus and vector by sailing [6]. Between 1939 and 1945, that pattern eventually changed, possibly with the impact of the Second World War (WWII). Dengue spread dramatically, especially to Southeast Asia and the Pacific Islands, due to the movement of troops and war equipment that enabled the transport of the virus and its mosquito vector [18]. By the end of WWII, many Asian countries became hyperendemic, with all the four serotypes in co-circulation [18,19]. WWII was therefore the driving force of the dengue pandemic. Following this historical event, a fast population growth period led to the increase of urbanization and globalization, together with the occurrence of hemorrhagic dengue fever epidemics, namely in Philippines (1953–1954) and Thailand (1958), followed by Singapore, Malaysia, and Vietnam (in the 1960s), and Indonesia and Burma (Myanmar) (in the 1970s) [19]. A broad-scale campaign by the Pan American Health Organization (PAHO) aimed to eliminate dengue vector. For about 20 years, dengue was absent. However, in 1964 a dengue epidemic occurred, with dengue re-introduced in the Pacific Islands and, in 1971, in the Americas [18,19]. Until recently, outbreaks have kept on occurring, especially in Latin America and Southeast Asia, as reviewed elsewhere [20]. So far, PAHO has reported more than 1.5 million suspected and confirmed cases in the Americas and Caribbean and more than 150,000 cases were reported from Asian countries [20]. According to the World Health Organization (WHO), in January 2020, a dengue epidemic was declared in the French Caribbean territories of Guadalupe and Saint-Martin [21,22].

Overall, dengue transmission has increased dramatically in the second half of the 20th century, with a global resurgence of dengue fever and the emergence of dengue hemorrhagic fever. These events seem to be related with demographic and society changes, namely global population growth, urbanization, lack of effective mosquito control where dengue is endemic, and increased air travel [13,23]. Dengue spread can be associated with the geographic spread and domestication of its vectors [18]. Consequently, the number of dengue cases reported to the WHO increased from 505,430 in 2000 to 2.4 million in 2010 and 4.2 million in 2015. In 2019, the largest number of dengue cases globally was reported [24]. It is clear that such a trend, accompanied by climate change and globalization of trade and travel, could result in a similar increase in the occurrence of dengue in Europe as well. Moreover, the number of deaths per year caused by this disease is estimated at approximately 20,000 [25].

Worsening the situation, the current SARS-CoV-2/COVID-19 pandemic is overwhelming the health systems, frequently in countries where dengue is endemic, such as Brazil [26], leading to a delay in dengue and/or COVID-19 diagnosis and subsequent treatment, which may contribute to an increased transmission and, possibly, number of deaths, despite the underreporting of the real number of cases and deaths. Dengue and COVID-19 share clinical manifestations, namely the febrile symptoms and rash, and is a confounding factor in diagnosing either disease [24,26,27,28]. In Singapore, two patients presented a false-positive result for dengue, being later tested for SARS-CoV-2 with positive results, delaying the result of COVID-19 and appropriate management. This highlights the importance of considering the possibility of false-positive dengue serological results [29]. Moreover, with the effort and measures to control COVID-19 transmission, some strategies to control dengue vector stopped. Since controlling mosquito populations remains the most effective prophylactic measure, this may lead to an increase in dengue cases in the future [30].

### 2.2. Dengue History and Epidemiology in Europe

**Figure 1:**
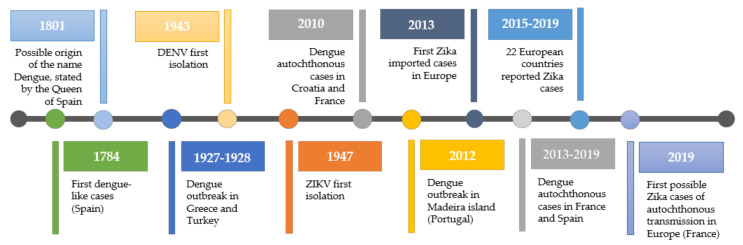
Timeline of dengue and Zika cases in Europe. Dengue has been present in Europe and is considered an emerging threat by the European Center for Disease Control and by the European Union (EU) public health authorities. The mosquito is found in the region, leading to recent outbreaks. Thus, it must be accounted for in terms of public health policies across the EU.

As mentioned above, the first documented dengue-like case in Europe dates back to 1784, in Spain [31,32,33]. During the 18th and 19th century, several epidemics occurred in eastern Mediterranean countries, mainly in ports, with the dengue vector broadly established in Southern Europe [31,33]. The largest dengue outbreak in the Mediterranean region occurred in 1927–1928, in Greece and Turkey, affecting more than one million people. Until 1930, dengue fever was endemic in those countries, due to the presence of *A. aegypti* [31,34,35]. The 1927–1928 Greek outbreak resulted in more than 90% of Athens’ population becoming sick. Recent investigations have shown that the high occurrence of dengue hemorrhagic fever was due to a sequential and almost simultaneous exposure to DENV types 1 and 2, in a previously unexposed population, causing the massive number of serious cases [31,34,35]. A high number of displaced people in Athens (more than 68% of the population being recent refugees fleeing as result of the defeat of the Kingdom of Greece against the Republic of Turkey and the subsequent forced population exchange) was living in squalid conditions, without running water (water storage receptacles may have helped mosquito spread) [31,34,35]. This provides valuable lessons for Europe: Exposure to the viruses in the presence of the mosquito can lead to serious epidemics, especially in fragile previously unexposed populations. Anti-mosquito campaigns plus access to running water helped contain further epidemics, alongside colder periods that also checked mosquito populations [31,34,35]. However, Europe is not protected from new outbreaks: Globalization of trade and travel, plus climate changes, can easily reverse the situation, especially in warmer regions, such as the Mediterranean basin. Recently, autochthonous dengue cases were reported in Croatia and France in 2010 [36,37]. In 2012–2013, the Autonomous Region of Madeira (Portugal) experienced a dengue outbreak with 1080 confirmed cases, being the major European outbreak since 1928. About 80 cases were confirmed in other European countries originated from people returning from Madeira [38,39,40]. Recently, the first seroprevalence study was conducted with a representative number of samples from the Madeira population, demonstrating that it is very likely that the number of cases before, during, or after the 2012 dengue epidemic was underestimated (7.8% of the population were seropositive in a representative sample of 237, while the reported infection rate in 2012 was 0.4%) [41]. Between 2013 and 2015, a total of 13 autochthonous cases of dengue were reported in France [42,43]. In 2018, France and Spain reported, respectively, eight and six locally dengue cases. In 2019, France and Spain reported again locally acquired dengue cases (nine and two, respectively) [43]. Of note, dengue is not endemic in Europe and most cases are travel-associated, with the same pattern being observed for Zika infection (Figure 1) (Table 1) [44]. In Table 1, it is easy to follow the impact of dengue and Zika in Europe, which are clearly mainly travel-associated cases (Table 1). Still, as shown, sporadic autochthonous infections do occur.

### 2.3. Zika History and Epidemiology Worldwide

Zika virus recorded history begins later. It was first isolated in 1947 from a rhesus monkey of the Zika Forest, in Uganda, near the shore of Lake Victoria, during studies to identify the vector of yellow fever [45]. In 1952, the ability of ZIKV to infect humans was evidenced by detecting the presence of neutralizing antibodies to Zika virus, in Uganda and Tanzania [46,47,48]. In 1954, the virus was isolated from a 10-year old girl in Eastern Nigeria [49]. While, for some authors, it is considered the first reported case of Zika virus in humans, other believe that the first reported case occurred in 1962, in Uganda [50,51]. In 1977, ZIKV human infection was reported outside Africa, in Indonesia. Until the 2000s, few human infection cases were reported (less than 20), being geographically limited, and epidemics were never reported. It is possible that periodic cases of Zika infection occurred, but they were misdiagnosed as other illness, such as dengue [7,47,52,53]. Between 1950 and 1970, serological surveys were performed, and the results suggest that the virus was widely spread across Africa and Asia [7,53]. In 2007, the first large outbreak was reported in the Yap islands (Federated States of Micronesia) [54]. This epidemic was followed by a larger one occurring in 2013–2014, in the French Polynesia [55]. During this epidemic, the Guillain-Barré syndrome, the most common and severe acute paralytic neuropathy, was linked to Zika infection [56,57,58,59,60]. That epidemic was followed by smaller outbreaks across the Pacific [60]. In 2015, Zika virus infection cases emerged in Brazil, but it is thought that the virus was introduced before [61]. During this epidemic, Brazil reported an association with Guillain-Barré syndrome in adults and microcephaly in newborns, a rare neurological condition where the head is smaller than normal for the child’s age and gender [47]. Subsequently, Zika virus had a rapid spread throughout Brazil and the Americas [8] and, in 2016, the WHO declared that Zika infection associated with microcephaly and other neurological disorders constituted a Public Health Emergency of International Concern (PHEIC) [8,47]. Asian countries, namely Singapore, Vietnam, and Thailand, have reported outbreaks since 2007. In 2015–2016, Cape Verde reported the first African Zika epidemic [53].

### 2.4. Zika History and Epidemiology in Europe

The first reported imported case of Zika occurred in Europe in 2013 [7,62]. According to the European Centre for Disease Prevention and Control (ECDC) data, between 2015 and the middle of 2019 no vector-borne local transmission of ZIKV was recorded. Yet, in the same period, 22 countries of the European Union/European Economic Area Member States reported ZIKV cases, either travel-associated or locally (but not vector-borne) acquired. The non-vector-borne transmission included sexual transmission and mother-to-child transmission (Table 1) [44,63]. In October 2019, France reported three likely cases of vector-borne transmission of ZIKV (Figure 1) [63,64,65]. Summing up, since the first large outbreak in Yap islands, in 2007, Zika virus has been dramatically expanding throughout the world, with a clear potential to be established in Europe, where mosquito vector populations are present [66].

## 3. The *Flavivirus* Genus

**Figure 2:**
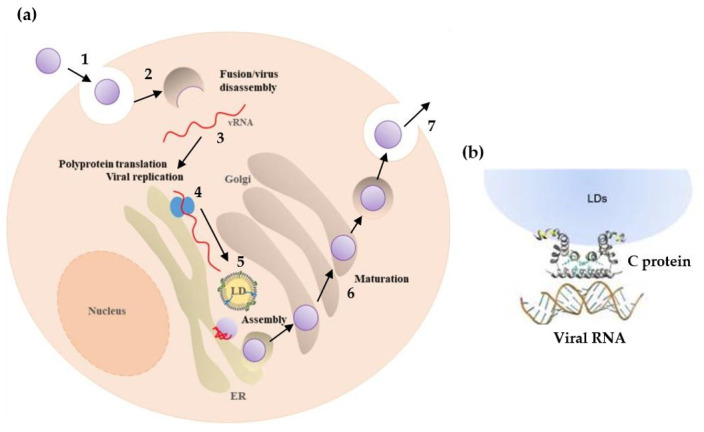
Flavivirus life cycle, highlighting the capsid protein role. (**a**) Flavivirus life cycle. The virion enters the host cell by clathrin-mediated endocytosis (1). Fusion of the viral envelope and the cell membrane is promoted by acidification of the endosome (2), followed by the viral genome release into the cytoplasm (3). The viral genome is translated into a polyprotein that is cleaved into 10 proteins: three structural and seven non-structural (4). Next, replication occurs surrounding the ER and LDs (5). This process is followed by virus packaging and assembly, to form new infectious viral particles (6) that follow a secretory pathway, being released through exocytosis (7). (**b**) The C protein interacts with host LDs and the viral genome, interactions that are crucial for DENV replication and genome packaging, respectively. Those interactions might be targeted in future therapeutic strategies. Adapted from [69,76].

The *Flavivirus* genus belongs to the *Flaviviridae* family and is composed by 53 species, including DENV, ZIKV, yellow fever (YFV), West Nile (WNV), and Japanese encephalitis (JEV) viruses. It is characterized by enveloped virions with icosahedral symmetry, containing a single-stranded positive-sense RNA genome (ss(+)RNA) (approximately 11 kb) with a single open reading frame flanked by 5’ and 3’ untranslated regions, with a 5’CAP and not poly-adenylated in the 3’-terminus [67,68]. The flaviviruses life cycle can be divided into seven sequential steps (Figure 2). First, the virion recognizes and attaches to receptor molecules on the host cell surface [67,69,70,71,72,73,74]. This process is dependent on the viral envelope (E) that binds to attachment factors such as glycosaminoglycans (e.g., heparan-sulfate proteoglycans and syndecans), concentrating the viral particles at the cell surface, followed by the interaction with primary receptors (e.g., αVβ3 integrins and C-type lectin receptors) [73,74]. This allows the virus entry into the cell through clathrin-dependent endocytosis. All this process mediated by the viral envelope (E) protein, which undergoes irreversible conformational reorganization as the endocytic vesicles become more acidic, promotes the viral envelope fusion with the host vesicle membrane, followed by viral RNA genome release into the cytoplasm. The viral genome is translated into a polyprotein, which is processed with the help of the host cell machinery into ten viral proteins. Three of these are structural proteins, the capsid (C), membrane precursor (prM) and envelope (E) proteins, while seven are non-structural (NS) proteins (NS1, NS2a, NS2b, NS3, NS4a, NS4b, and NS5). Next, replication occurs on virus-induced host cell membranes, which are associated with the replication complex, near the endoplasmic reticulum (ER) and lipid droplets (LDs). A positive (+) strand genomic RNA is used as a template to originate a complementary negative (-) RNA, resulting in a double strand (ds) RNA intermediate (termed replicative form). The (-) RNA strand within the intermediate form serves as template for the synthesis of new molecules of positive (+) strand RNA [75]. Viral genome copies are associated with the C protein and packaged in a process involving ER-derived membranes containing prM and E viral proteins. Then, the virion follows the cellular secretory pathway, causing the maturation of the virion in the trans-Golgi network (where prM is cleaved into membrane protein (M) through a furin-mediated process). Finally, the new infectious particles are released into the extracellular medium, by exocytosis [67,69,70,71,72,73,74].

### 3.1. The Flavivirus Proteome

**Figure 3:**
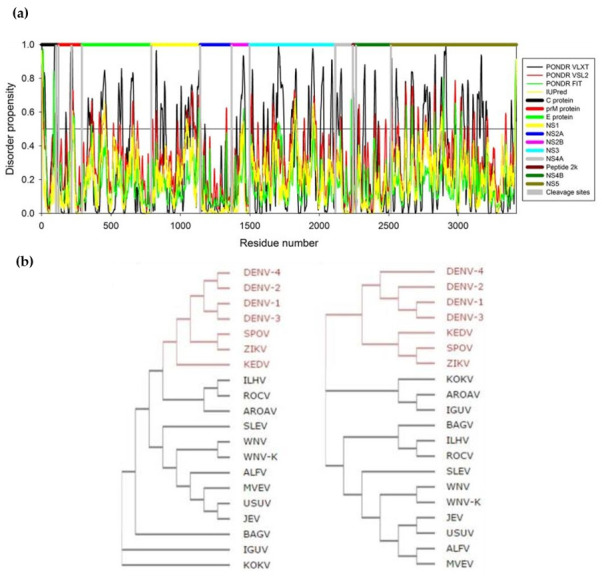
*Flavivirus* polyprotein disorder propensity. (**a**) The *Flavivirus* proteome is rich in structurally disordered regions (values scored above 0.5 in the graph). (**b**) Flaviviruses use disordered protein regions to extract more function out of a small genome. One such proteins is the C protein, which amino acid residues sequence variability (left) is representative of that of the polyprotein (right). The same clades within mosquito-borne flaviviruses are observed. Abbreviations: SPOV—Spondweni virus; KEDV—Kedougou virus; ILHV—Ilheus virus; ROCV—Rocio virus; AROAV—Aroa virus; SLEV—Saint Louis encephalitis virus; WNV-K—WNV serotype Kunjin; ALFV—Alfuy virus; MVEV—Murray Valley encephalitis virus; USUV—Usutu virus; BAGV—Bagaza virus; IGUV—Iguape virus; KOKV—Kokobera virus. Adapted with permission from [76,77,78].

The flaviviruses polyprotein is very rich in disordered regions [77,78], with the C protein being an excellent tracker of *Flavivirus* spp. phylogeny [76] (Figure 3). This abundance of intrinsically disordered (IDP) regions (Figure 3a) with a promiscuous binding activity enables multiple functions, as observed for the particularly relevant C protein. Tracking the variation of amino acid sequences within the polyprotein as a whole or only within the C protein results in similar clades of mosquito-borne *Flavivirus* spp. viruses (Figure 3b). The C protein is thus a good indicator of viral evolution. These disordered regions enable flavivirus small proteomes to have a large interactome, enabling multiple functions [78,79]. Flaviviruses are a paradigm for the role of IDPs in increasing viral functions. This understanding is of particular importance, as it helps to design therapeutics to their key regions and proteins, namely the C protein, described below.

### 3.2. The Flavivirus C Proteins

The capsid protein is better described here due to its key roles in the viral life cycle and interaction with host cell machinery, as well as being one line of research in our laboratory. *Flaviviridae* C proteins are structural proteins that, conjugated with the viral RNA, to form the nucleocapsid [67,70,80,81,82]. Deletion of the DENV C coding region results in empty viral particles, highlighting the role of this protein in viral assembly and encapsidation [83]. From a structural point of view, the C proteins from the *Flavivirus* genus are relatively homogeneous, having around 100 amino acid residues and forming homodimers in solution [80,81,82]. There is an asymmetric charge distribution, with most positive charges of DENV C concentrated in one side of the dimer, formed by the α4 helices of each monomer (α4-α4’), while the opposite side has a higher density of non-polar residues. This is intimately related with the *Flavivirus* C proteins’ function(s), with the α4-α4’ region being likely responsible for interaction with the viral RNA, assisting in the viral genome packaging, whereas the α2-α2’ region interacts with host lipid systems (Figure 2b) [69,76,78,81,84,85].

Individuals infected with *Flaviviridae* frequently have liver involvement that leads to liver steatosis (known as “fatty liver”) [86,87,88,89], with an increased number and size of lipid droplets (LDs) in hepatocytes and liver macrophages (Kuppfer cells) [88]. LDs are cytosolic structures made of neutral lipids (triacylglycerols and cholesterol esters), polar lipids (phospholipids and cholesterol), and specific proteins, essential for the cellular lipid homeostasis, storing, and distributing lipids between other organelles [90,91]. In DENV-infected cells, when the formation of these structures is inhibited (or LDs homeostasis is affected), the infectious virus titers significantly decrease [92,93,94]. It was also found that DENV C interacts with host cell LDs and that this interaction is crucial for DENV replication [69,92]. These findings demonstrate a clear connection between host lipid metabolism and the C protein. Additionally, the interaction with LDs involves residues conserved between *Flavivirus* C proteins, from the α2-α2’ domain and from the disordered N-terminal region (namely the region encompassing DENV C amino acid residues 14 to 23) [92,95].

All of this led to the design and patenting of a new drug lead, pep14-23, which inhibits DENV C-LDs interaction [95,96,97]. Furthermore, DENV C IDP region has a motif similar to importin α, a protein involved in nuclear transport and signaling, which may allow DENV C binding to importin β [76,97]. This motif is conserved among mosquito-borne flaviviruses, suggesting a key role [95,98]. In fact, ivermectin, an inhibitor of importin α/β mediated transport, inhibits YFV, DENV, and JEV in vitro replication [99,100]. Those interactions, where a viral process depends on the host cell machinery and specific regions of proteins are involved, constitute promising targets for therapies, which will be discussed further below.

## 4. Pathogenesis of DENV and ZIKV Infections

**Figure 4:**
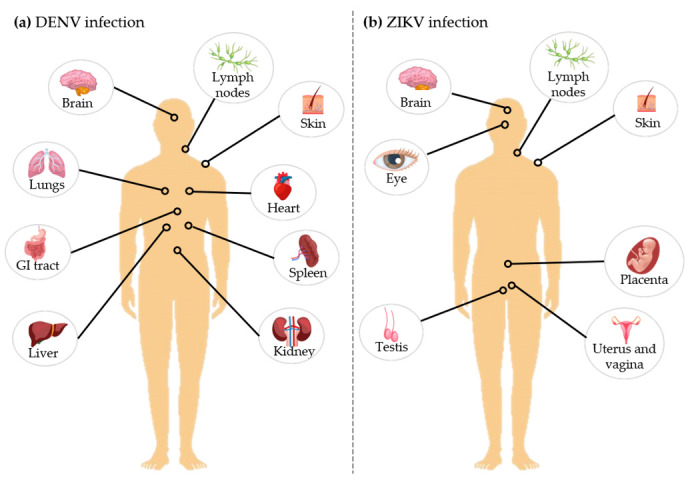
Dengue and Zika viruses tissue tropism. Representative target-organs of (**a**) DENV and (**b**) ZIKV are highlighted. This image has been designed using resources from Freepik.com.

The ability of a virus to infect specific types of cells and tissues (tropism) may provide clues about targets to develop antiviral treatments, based on that specificity. At the mosquito bite/feeding event, both DENV and ZIKV are transmitted through the mosquito saliva into the bloodstream of the host, firstly infecting nearby skin cells (e.g., keratinocytes and Langerhans cells). Next, the virus spreads to the lymph nodes, followed by the dissemination to other tissues and organs. Tropism for cells from the liver, immune system, endothelium (blood vessels), and retinal cells has been described for DENV [101,102,103]. Additionally, at autopsy, DENV was detected in the human brain, thymus, lung, bone marrow, kidney, lymph nodes, spleen, liver, gastrointestinal tract, heart, and skin (Figure 4a) [101]. However, some of these target cells or tissue roles in viral dissemination are still controversial [101].

ZIKV is able to infect monocytes, which can infiltrate into tissues such as the brain and placenta, being used as a "Trojan horse". Besides those organs, ZIKV has been detected in the eye, testis, uterus, and vagina, as well as in body fluids (Figure 4b) [104,105,106,107]. In vitro and in vivo studies are being done to unravel other cells and tissues that might be permissive to ZIKV and DENV infection, and to explore their role in viral dissemination. The cell and tissue tropism of those viruses might be correlated, at some level, with the clinical outcome, as described below.

### 4.1. Dengue Infection

**Figure 5:**
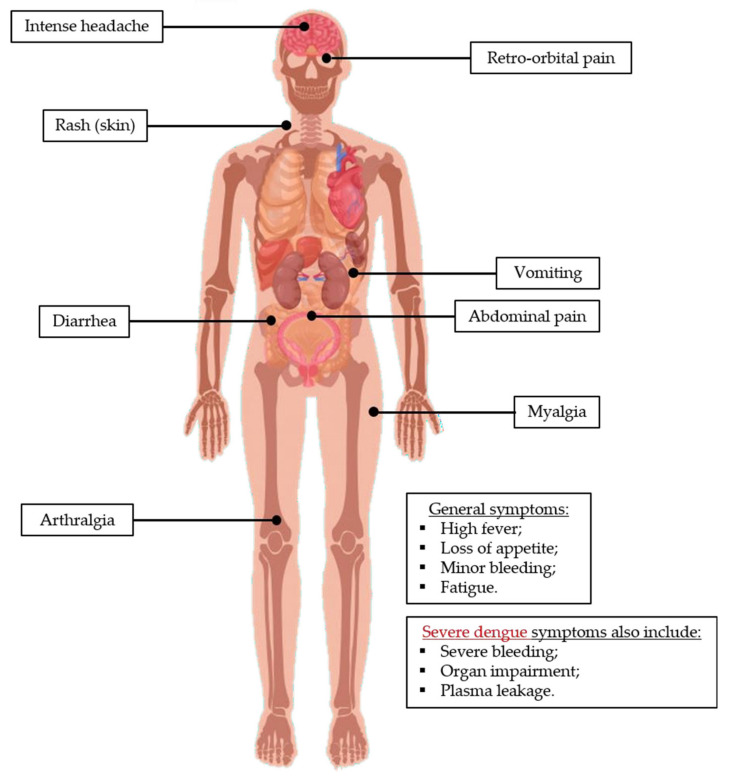
Dengue infection clinical symptoms. The typical clinical manifestations of dengue include flu-like symptoms, namely headache, fever, and fatigue, plus a number of more specific symptoms such as rash and severe myalgia and arthralgia. When it evolves to severe cases, it can induce bleeding, organ impairment, and loss of fluids, all of which may lead to death. This image has been designed using resources from Freepik.com.

Dengue infection causes a systemic disease characterized by a wide clinical spectrum (Figure 5), with up to 40–80% of asymptomatic cases [108,109,110]. Symptomatic cases can be mild or severe (Figure 5). According to the WHO Dengue classification of 1997, symptomatic dengue infections were classified into three categories: undifferentiated fever, dengue fever (DF), and dengue hemorrhagic fever (DHF). The latter was divided into four severity grades, with grades III and IV representing dengue shock syndrome (DSS), which can often be fatal. In 2009, a new classification was proposed, where the non-severe cases were split into two groups: without warning signs and with warning signs of potential severe dengue [111]. After infection, an incubation period of 3 to 14 days occurs. Symptomatic infections can be characterized by high fever, intense headache, retro-orbital pain, loss of appetite, vomiting, diarrhea, abdominal pain, rash, minor bleeding (nose or gums), extreme fatigue, and intense arthralgia and myalgia, explaining the popular designation of the disease as “break-bone fever” (Figure 5) [12,15,24,112,113,114]. Occasionally (<5% of the cases), the disease progresses to severe dengue (including DHF and DSS). This is defined by the presence of “classic” symptoms of dengue with any of the following conditions: severe bleeding, severe organ impairment, or severe plasma leakage leading to fluid accumulation with respiratory distress, which can be a life-threatening condition (Figure 5) [12,110,112,114,115,116,117].

Concerning DENV, there are four major serotypes (DENV-1 to DENV-4) and, within each one, three to five common genotypes (strains), each with variable degrees of virulence. The interaction of several factors, such as virus strain, genetic background of the host, and immune response to previous dengue infections, strongly influences the outcome and severity of the disease [118,119]. Predicting the outcome of an infection is therefore difficult but, it is clear that there is an increased risk to develop severe dengue in a second DENV infection, by a different serotype [120]. This is consistent with the hypothesis of antibody-dependent enhancement of infection, leading to a cytokine storm: when an infection with a different DENV serotype occurs, antibodies from a previous infection contribute to exacerbate the symptoms [102,117,120,121,122]. This can also occur in children with passive antibodies transfer from the mother during gestation [102,117,120,121]. This is particularly worrisome in regions where multiple DENV serotypes are co-circulating. In agreement with that, a study concluded that severity is correlated with high viremia titers, secondary infection, and DENV-2 serotype [123].

### 4.2. Zika Infection

**Figure 6:**
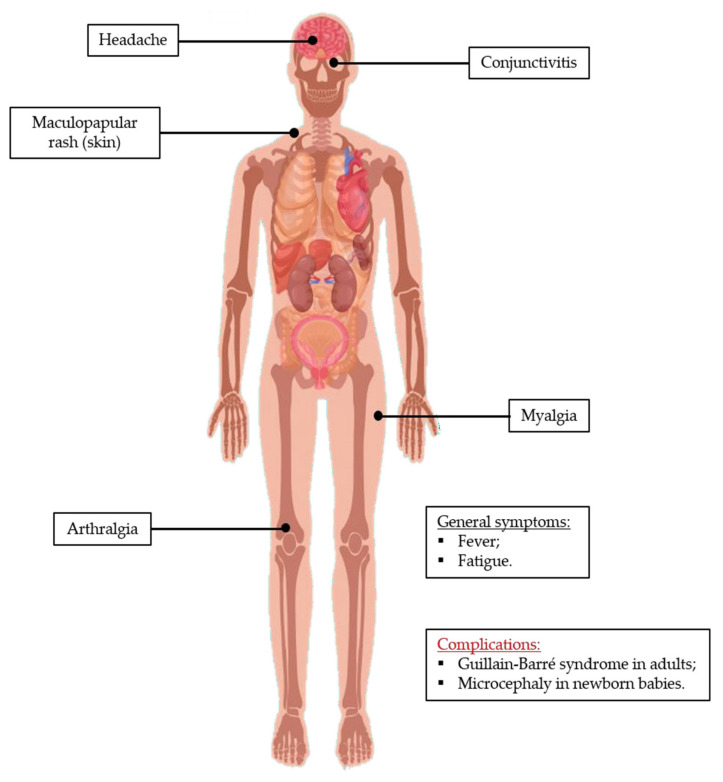
Zika infection clinical symptoms. The typical clinical manifestations of Zika include flu-like symptoms, namely headache, fever, and fatigue. Myalgia, arthralgia, and conjunctivitis are also commonly observed symptoms. However, the most serious complications are the development of Guillain-Barré syndrome in adults and microcephalia in newborn babies, which are devastating consequences of Zika infection. This image has been designed using resources from Freepik.com.

Reports of widespread ZIKV infection are more recent and there are fewer epidemic data available, when compared to DENV. However, literature on ZIKV infection shows that the spectrum of reported clinical manifestations of the infection (Figure 6) has been increasing. As with dengue infection, many people infected with ZIKV are asymptomatic (about 80%), and a period of incubation between 3 to 14 days occurs. Typical symptoms are fever, headache, conjunctivitis, maculopapular rash, fatigue, arthralgia, and myalgia. Those usually last up to 7 days. A small fraction of infections may result in more complicated clinical outcomes. Zika virus infection has been associated with Guillain-Barré syndrome in adults, and microcephaly and other severe fetal brain defects in newborn babies (Figure 6) [7,9,124].

Phylogenetic analysis identified three ZIKV lineages: one Asian and two African [9,125], the first being responsible for the most recent serious outbreaks [9,126]. A study published this year reports that an African lineage of Zika started circulating in Brazil (probably since 2019), possibly causing a new epidemic [127]. This shows how well the virus circulates through the planet, and how even regions already affected can suffer new epidemics from different viral lineages (up to then, Brazil had only contacted with the Asian lineage of Zika). The easy spread of viral strains/lineages not only results in new epidemics, but can also, as in dengue, cause cross-reactions that may eventually lead to secondary infections by different strains, making future outbreaks potentially more severe. Questions about which lineage is most virulent and/or causes neurological complications have been arising, but the answers are unresolved. In vitro, ex vivo, and in vivo studies suggest that ZIKV African lineage strains are intrinsically more virulent than Asian ones [126,128,129]. However, it is still not clear if those differences materialize themselves in the observed diverse clinical manifestations. Simonin et al. speculated that Asian lineage strains may result in infections within the central nervous system of fetuses, while African lineage strains may result in acute infection [126].

## 5. Dengue and Zika Diagnosis

Early laboratory diagnosis can lead to better management. Less severe viral diseases promote similar undifferentiated symptoms. A portion of symptomatic patients can progress to DHF and the much more serious DSS, and a correct diagnosis is essential. Molecular, biochemical, and serological methods can be performed to test DENV infection, namely virus isolation and serotype identification, nucleic acid detection, antigen detection (e.g., DENV NS1 protein), IgM and IgG seroconversion, and detection of IgM and/or IgG levels in acute serum [130,131]. The latter does not confirm a dengue infection but suggests a probable case. Virus isolation provides the most specific result. Nucleic acid and viral antigen detection are more practical; alongside serological assays of anti-DENV antibodies, either can confirm DENV infection [130,131]. Routine laboratory confirmation is frequently unaffordable in tropical and sub-tropical developing countries, the most affected by dengue. These are mostly regions of the planet with high poverty levels, highly populated cities, and weaker health systems. Thus, many dengue infections may be unreported, with the diagnosis being mostly or solely based on the patient clinical history, leading to poor estimates of dengue cases, hospitalizations, and deaths [131,132].

Laboratory methods to diagnose ZIKV infection include virus isolation, viral RNA detection with molecular assays, antigen detection, and detection of antibodies against ZIKV. There are three USA Food and Drug Administration (FDA)-authorized diagnostic tests to detect ZIKV antibodies, all approved in 2019: ZIKV Detect 2.0 IgM Capture ELISA, ADVIA Centaur Zika test, and LIAISON XL Zika Capture IgM Assay II [7,104,133]. Serological tests are not ideal, as cross-reactivity with other members of *Flavivirus* genus may lead to a misdiagnosis [7,104,131]. This was also found for DENV, being a confounding factor for the estimates of these flaviviruses infection cases [7,104,130,131]. Very recently, nanosensors (based in gold nanorods functionalized with DENV envelope protein) were developed for DENV infection immunodiagnostics, and were demonstrated to be able not only to distinguish the DENV serotype responsible for the infection, but also to discriminate between DENV and other flaviviruses, such as ZIKV, thus avoiding the frequently observed cross-reactivity [134]. This is based on the optical feature of gold nanoparticles, the localized surface plasmon resonance (oscillation of electrons in the particle surface upon irradiation with light). Therefore, any changes occurring close to the nanoparticle result in modifications in the absorption spectra. In this specific case, the detection of antibodies is accomplished by analyzing modifications in the light absorption wavelengths of the nanosensor [134].

## 6. Dengue and Zika Treatments/Vaccines

### 6.1. Clinically Approved Treatments/Vaccines

DENV infection treatments are focused on alleviating symptoms, especially at earlier stages. These include body temperature control to reduce fever (antipyretics), decreasing pain (through analgesics), and allowing the body to heal. Non-steroidal anti-inflammatory drugs should not be used. In DHF and DSS, maintaining body fluids is critical. These non-specific therapies require medical doctors to be aware of the diagnosis [24]. According to the FDA, there are no approved vaccines or other specific treatments for ZIKV infection [133]. ZIKV symptoms are treated with drugs such as paracetamol to reduce fever and pain, antihistamines for pruritic rash, alongside ingestion of fluids, to avoid dehydration [7,135,136]. Aspirin and non-steroidal anti-inflammatory drugs (NSAIDs) are not recommended, due to increased risk of hemorrhagic syndrome in flavivirus infections and Reye’s syndrome in children and teenagers [7,135,136]. A recent study, however, found that ibuprofen was frequently used as an over the counter drug to treat pain/fever (up to 1,200 mg/day for up to 10 days), and minimally increased bleeding risk [137]. Statistically significant increases (in bleeding incidence and/or volume) were reported but were not clinically relevant. The authors suggest that ibuprofen at these doses is relatively safe and that recommendations against NSAID in dengue treatment should be reassessed. Nevertheless, further studies should be completed to validate such approach and fully weight its risks and benefits.

Having specific treatments would be ideal. That is not easy, namely for ZIKV infection treatment, as any drug must not only be efficient and safe, but also able to cross the placental barrier and the blood–brain barrier. This is a big challenge that, until now, has not been met, as no drug has been found that is clinically safe and efficient for that purpose (in spite of some promising advances, detailed ahead). Concerning vaccines, Dengvaxia (CYD-TDV), developed by Sanofi Pasteur, is the first and only licensed dengue vaccine. It has been approved by regulatory identities in 20 countries, including FDA in 2019 and the European Medicines Agency (EMA) in 2018 [24,138,139,140,141]. However, its efficacy varies according to DENV serotype, serostatus (status of having detectable dengue antibodies), and age [24,138]. According to the WHO, the vaccine is recommended to people living in regions where dengue is endemic, from 9 to 45 years old, and who have had at least one previous DENV infection [24].

### 6.2. Treatments/Vaccines Under Development

Other vaccines are under clinical trials. For example, TAK003 (Takeda Pharmaceutical Company) is currently undergoing phase III clinical trials (NCT02747927) [121,138,142]. This vaccine candidate is a live-attenuated tetravalent dengue vaccine. Recent data of the clinical trials demonstrated that the vaccine induced a long-term immune response, reducing the risk of symptomatic disease regardless of the person serostatus [143,144]. For ZIKV, no vaccines are available, but several candidates are under development, including in clinical trials [133,145,146]. One of those candidates, VRC 705 (National Institute of Allergy and Infectious Diseases/Vaccine Research Center), elicits good immunogenic response, now in phase II clinical trial (NCT03110770) [145,146,147]. This is certainly a promising step in the right direction.

Different steps of the viral life cycle are also being targeted, via the development of new drugs, alongside the screening of compound libraries and the repurposing of existing drugs. Natural products and antibody-based candidates are under evaluation. Antiviral drugs may target not only the virus, but also key host cell mechanisms [104,136]. Actually, targeting host factors might be a more interesting strategy to avoid viral evolution and resistance [148]. All cards are on the table and under evaluation, as discussed below. Regarding antiviral drugs, the main targets that are being studied for both viruses are NS3, NS4B, and NS5 [136,149,150,151]. Several potential antivirals against those targets have been tested at least in vitro, as described elsewhere [136,152]. Of note, BCX4430 (galidesivir), a selective inhibitor of RNA-dependent RNA polymerase (RdRp, within NS5) [136], is undergoing phase I clinical trials to evaluate safety, pharmacokinetics, and antiviral activity in subjects infected with yellow fever virus and SARS-CoV-2 [153]. The antiviral activity of this compound against ZIKV has already been tested in cell culture and in a mouse model of severe ZIKV infection, demonstrating a favorable result in an improved outcome [154]. More recently, a study was conducted administrating galidesivir to ZIKV infected rhesus macaques. The drug demonstrated to be safe and conferred post-exposure protection against ZIKV infection [155]. Moreover, sofosbuvir, an FDA-approved RdRp inhibitor for hepatitis C virus, displayed in vitro antiviral activity against DENV and ZIKV, protecting mice from ZIKV-induced death [152,156,157].

The development of strategies to inhibit flaviviruses C proteins may also be worthy, as C proteins from other virus (e.g., human immunodeficiency virus) have already been proven to be valid as target for antiviral drugs [148,158]. This strategy is particularly focused on inhibiting C protein interactions with host and/or viral elements [148]. Regarding antiviral drugs peptidomimetics focusing on the C protein, we designed and patented a promising drug lead, pep14-23. This peptide is based on a conserved segment comprising amino acid residues 14 to 23 of DENV C. In vitro studies demonstrated that it inhibits the interaction of DENV C with host intracellular LDs, an interaction essential for viral replication [95,96,97,98]. Given C protein similarities among *Flavivirus*, pep14-23 will be tested against ZIKV C and closely related pathogens. Concerning other targets, apolipoprotein E (APOE), the target of DENV C on very low-density lipoproteins (VLDL), contains an α-helical N-terminal sequence/structure that superimposes with DENV C α-helices 1 and 2. DENV C hydrophobic pocket accommodates APOE α-helix 4 [98,159]. Taking this into consideration, other strategies are under development, namely, APOE-based peptides based in those α-helical regions, which may act as inhibitors of *Flavivirus* C proteins. Other antivirals against C proteins have been exploited, such as ST-148, an inhibitor of DENV C that binds the hydrophobic pocket between the monomers, stabilizing C protein self-interaction (dimerization), which reduces viremia [160]. The same approach may be applied to ZIKV [150]. Mutations introduced within the capsid gene lead to the production of viral particles that are immunogenic but defective in packaging the viral genome (sub-viral particles) [148]. This may provide a good basis for the development of live-attenuated vaccines. In fact, immunization of mice with a single dose of a vaccine containing a mutated ZIKV capsid gene conferred protection upon challenge with ZIKV and protected against vertical transmission of infection [161]. On the same basis, data obtained from assembly defective DENV virions (NS2 protein mutant) stimulated the development of vaccines based on those pseudo-infectious viruses, which are able to induce immunogenic response but are defective in viral assembly [162]. Immunization of mice with a single-dose was effective in preventing infection from a wild-type challenge [162]. Of course, one must use with care the definition of wild-type viruses, as wild RNA viruses commonly exist as a “swarm of viruses” with varying genomes [163]. These variations are due to: (*i*) lack of proof-reading capability in the virally encoded RNA-dependent RNA polymerase; (*ii*) production of a large number of viral genomes; and, (*iii*) the absence of RNA repair systems both in host eukaryotic cells and in viruses [163]. In a quasispecie, the wild type is not a specific sequence, but consists of the weighted average of nucleotides at each position [163]. Thus, it is evident that field tests in real populations are the true measure of the efficacy of any vaccine or treatment.

Regarding antibody-based therapies, a few monoclonal antibodies provided protection against ZIKV in mice [149,164]. One of those, ZIKV-117, displayed ZIKV specificity and protected from vertical transmission [164,165]. Human polyclonal antibodies produced in transchromossomal bovines were administrated to mice, offering protection against ZIKV lethality and tissue damage [166]. For DENV, a monoclonal antibody developed by Visterra (Cambridge, MA, USA), Ab513, was constructed to bind a specific domain of the E protein of all DENV serotypes [151]. This antibody was able to neutralize several DENV genotypes and ameliorate dengue disease outcomes in mice (e.g., mitigates thrombocytopenia and vascular leakage, reduces viremia, and confers protection to mice fetus against antibody-dependent enhancement effect) [151,167]. Nevertheless, antibody-based therapeutics must be approached carefully, as antibody-dependent enhancement effect (described for DENV) may also occur for ZIKV [149,168]. Thus, epitopes causing that effect should be identified and avoided [164]. Otherwise, the desired protection would not be achieved and, instead, serious disease outcomes could even occur [149].

Flaviviruses, as for other viruses, require host cellular factors and machinery, manipulating the host cell to their own advantage [71]. Consequently, any molecule of any step of the viral life cycle can be a potential target for host-directed antivirals. These steps include the attachment of the virus to the host cell, entry into the cell, endosomal fusion, translation, replication, assembly, and maturation [149,150]. For example, chloroquine, a widely used anti-malarial drug, is proposed to interfere with viral entry. It has already been shown that it has anti-ZIKV activity [136,150]. However, in contrast to ZIKV, chloroquine did not show significant ability to reduce viremia in DENV-infected patients [151,169]. Upon viral infection, interferon (IFN) signaling pathways are induced [149,170]. Studies demonstrated that IFN-α, IFN-β, and IFN-γ inhibit ZIKV replication in vitro [149,171]. However, type I IFNs might be associated with pregnancy complications and, thus, must be used with great care [172]. Antiviral activity of IFN-α against DENV was also evaluated, showing that it inhibits viral replication in cell culture, as well as in mice [170]. Stimulation of immune response and/or viral targeting may also occur through microRNAs that modulate gene expression [173,174], or even acting at an epigenetic level [175]. Thus, several treatments may become viable clinical options in the future. Still, plenty of research and development are needed to ensure their efficacy and safety.

## 7. Interplay Between Dengue and Zika

ZIKV and DENV are antigenically related viruses, co-circulating in certain regions [176,177,178,179]. One consequence of this similarity is misdiagnosis, as cross-reactivity occurs within members of this genus. DENV and ZIKV periodic epidemics were studied attempting to find possible correlations. Dengue incidence in Brazil and Colombia was examined before, during, and after the 2015–2016 Zika epidemic. The study revealed that in 2017, after the ZIKV epidemic, dengue incidence was low, suggesting that cross-protection decreases dengue incidence after Zika outbreaks [180]. Another possibility is that, given the high profile Zika pandemic, anti-mosquito campaigns during and immediately after gained strong impetus and political support, thus becoming more effective than in other years. This low dengue incidence was followed by its resurgence, with a large increase [180]. Mosquito control could have lost some of its political support, not being a priority anymore (eventually due, paradoxically, to its success and the associated reduction in the number of cases), directly leading to the increased incidence. This alternative explanation, that we can call a man-made variation in the number of cases cannot, unfortunately, be discarded.

Another relevant aspect, related to ZIKV association with microcephaly (congenital Zika syndrome), is that it was found to have higher incidence in a specific Brazilian region [181]. An immediate possible explanation was that DENV-mediated immune enhancement could promote that neurological complication. However, this hypothesis was refuted [181]. DENV seroprevalence was significantly lower among ZIKV-seropositive mothers of children with congenital Zika syndrome (compared to ZIKV-seropositive control mothers), suggesting that DENV infection may even protect against the development of that syndrome [181]. In line with that, another study with pregnant women with possible ZIKV infection found three women who were DENV and ZIKV immunologically cross-reactive and another two women who were not [179]. The three women that were previously exposed to DENV did not vertically transmit ZIKV to the fetus, while the two (who were not exposed to DENV before) did transmit the virus vertically [179]. Once more, this suggests that previous contact with DENV confers cross-protection [179]. At an immunological level, DENV/ZIKV cross-reactive Th1 CD4+ T cells are able to suppress ZIKV replication in an antibody-independent manner [182]. It was also demonstrated that CD8+ T cells from a DENV infection mediate cross-protection to ZIKV infection [176]. Cross-protection against DENV has also been observed. Mice immunized with a ZIKV DNA vaccine candidate (pV-ZME) produced cross-reactive antibodies, cytokines, and CD8+ T cells response, leading to cross-protection against DENV1-4 [177]. However, in vitro studies demonstrated that ZIKV antibodies are able to exacerbate DENV infection [183]. According to this, it was observed that mouse pups with maternal antibodies against ZIKV, after DENV challenge, developed severe dengue. Therefore, maternal antibodies may influence the outcome of DENV infection in the offspring [184]. In vivo experiments using rhesus macaque came to the same conclusion: Previous ZIKV infection can enhance DENV infection [185]. Co-infection cases of DENV/ZIKV, DENV/Chikungunya virus, ZIKV/Chikungunya virus, or even triple virus co-infection have already been reported [186,187,188]. In epidemiological terms and for public health planning policies, it is thus important not only to consider what serotypes of DENV are co-circulating within a given region, but also if ZIKV is present, as cross-reactivity dramatically changes the effects of the infections, their gravity, and the burden to public health systems.

## 8. Mode of Transmission

**Figure 7:**
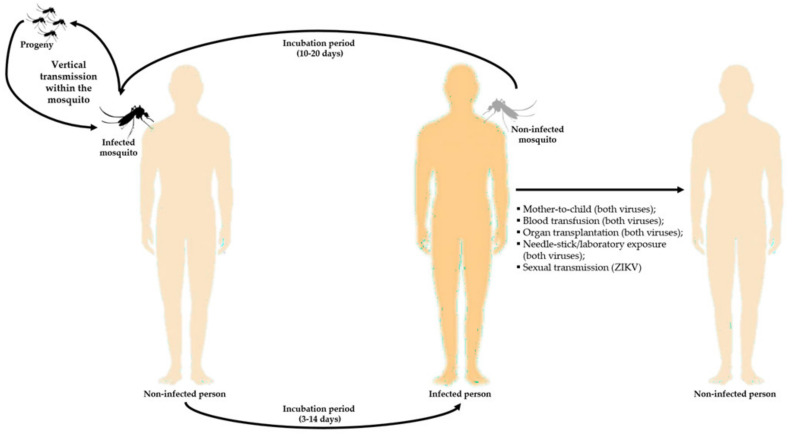
Modes of transmission of DENV and ZIKV. The main mode of transmission for both viruses is horizontal transmission (from infected mosquito to human). However, other modes have been suggested or reported, as well as vertical transmission (from an infected female mosquito to its progeny). This image has been designed using resources from Freepik.com.

Dengue and Zika are both arthropod-borne viruses (or arboviruses), mostly transmitted by *Aedes* spp. mosquitoes. DENV and ZIKV spread occurs mainly as horizontal transmission, thus from an infected vector to humans (Figure 7). However, vertical transmission within the vector population (from an infected female mosquito to its offspring) was also reported for *Aedes aegypti*, both experimentally and in natural conditions (Figure 7). This is thought to be a mechanism to ensure viral maintenance during unfavorable conditions (e.g., environmental limitations), without the apparent requirement of human infection [189,190,191]. Other modes of transmission of both viruses, even if rare, have already been described or suggested, namely from mother to child (vertical transmission), blood transfusion, organ transplantation, and needle-stick/laboratory exposure [192,193]. For ZIKV, sexual transmission has also been reported (Figure 7) [192].

The most effective transmission vectors are *A. aegypti* and *A. albopictus* female mosquitoes. These are now disseminated throughout tropical and sub-tropical regions, and adapted to urban environments, particularly *A. aegypti* [2,7,8,9,10]. That partially explains *A. aegypti* greater efficacy as transmission vector, since in urban environments DENV and ZIKV life cycles are sustained between human and vector organisms. The fact that *A. aegypti* feeds mostly on human blood also contributes to that hypothesis [10,194]. However, for sylvatic strain outbreaks, *A. albopictus* can be more efficient in transmitting the viruses as it feeds on a variety of mammals and avian species [195]. Sporadically, sylvatic strains can infect humans, causing DENV outbreaks, although those cases are usually less severe than the “standard” urban dengue infections [196,197,198]. For other flaviviruses, including ZIKV, sylvatic transmission cycles involving non-human primates and forest-dwelling mosquitoes also occur, suggesting that humans are not the only reservoir. Even though those viruses are adapted to the urban transmission cycle, this does not mean that sylvatic cycles are irrelevant. In fact, they may have a role in the re-emergence once a human epidemic has already disappeared; they might also afford selective environments, raising the possibility of development of new strains with a different degree of virulence for humans that even may overcome vaccine-derived immunity (in the case where vaccines are available) for the existing urban strains (vaccine redundancy) [199].

When the mosquito bites an infected person during feeding, viral particles enter the vector dietary tract and infect epithelial cells. Next, a period of incubation of approximately two weeks occur, when the virus disseminates through the mosquito, including in the salivary glands. Then, once the mosquito bites another person, it injects its saliva containing DENV or ZIKV viral particles, closing the cycle [66,200]. Immediate mechanical transmission, a mechanism consisting of the transfer of the viruses from an infected host to a susceptible one occurring in a short period between the two feeding events, may also occur [200,201]. Interestingly, *A. aegypti* mosquitoes are susceptible to DENV and ZIKV co-infection, influencing vector competence, with ZIKV being preferentially transmitted to the susceptible host [202].

### 8.1. Expansion of Mosquito Vectors

**Figure 8:**
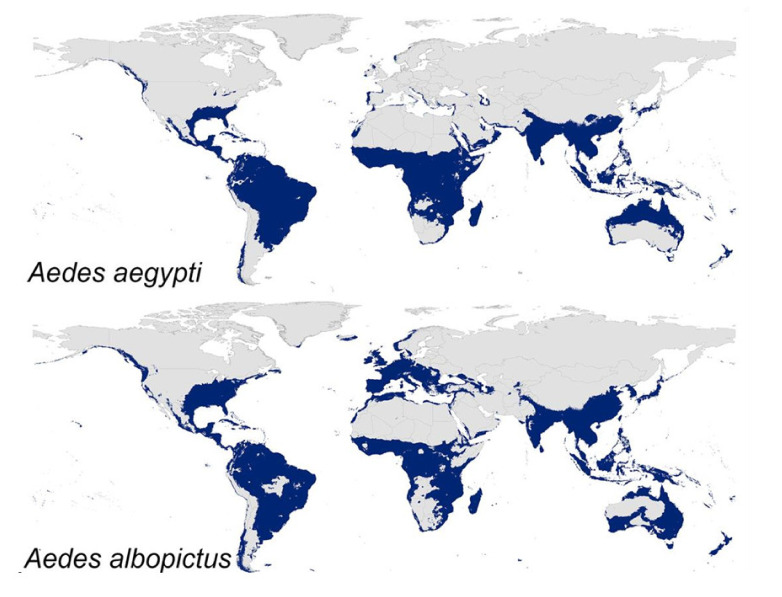
Potential distribution of *Aedes aegypti* and *Aedes albopictus*. This distribution is based on present climatic conditions. Blue shaded areas are projected as suitable and grey shaded regions are unfavorable. Adapted from [205].

During previous centuries, the native *A. aegypti* (from Africa) and *A. albopictus* (from Asia) adapted to exponential human population growth and occupancy of natural habitats [11,203]. These mosquitoes have undergone domestication, now being able to live in close proximity with humans and resort to anthropophagy. Consequently, mosquitoes can spread, along with humans, to other countries [11]. Moreover, viruses are also expected to have increased ability to be transmitted to humans, increasing their spread. This also results from viral adaptation due to co-evolution with their vectors and hosts, so they can be efficiently transmitted between them [11]. Expansion of vectors does not depend solely on environmental changes, but also on urbanization, human movements, and other socio-economic factors [11,203]. Of note, *A. albopictus* is more able to survive in colder regions (e.g., temperate regions) than *A. aegypti* and it is also well adapted to rural environment, where they are harder to control [203,204]. In fact, *A. aegypti* displays a potential wider distribution across the tropical and sub-tropical regions, while *A. albopictus* is also able to spread to more temperate regions (Figure 8) [205].

The first importation record of *A. albopictus* into Europe dates back to 1979, in Albania, where it became established. Approximately ten years later, it was found in Italy, being disseminated through different regions of the country [195]. Then, the mosquito was reported in France (1999) [206] and Belgium (2000) [207], but it was later eradicated. Since 2000, having become established in Albania, Italy, and Cote d’Azur (France), the mosquito has been expanding to other European countries, namely Greece and other Balkan countries [195]. The mosquito vector was also reported in Switzerland (2003) [208], Spain (2004) [209], the Netherlands (2005) [210], Germany (2007) [211], and Malta (2009) [212].

*A. albopictus* populations have been established in Slovenia (2007) [213], Russia (2011) [214], Turkey (2011) [215], and Romania (2012) [216]. Overall, its presence has been reported in a total of 23 European countries and 3 microstates, implying that their spread is continuing [195].

**Figure 9:**
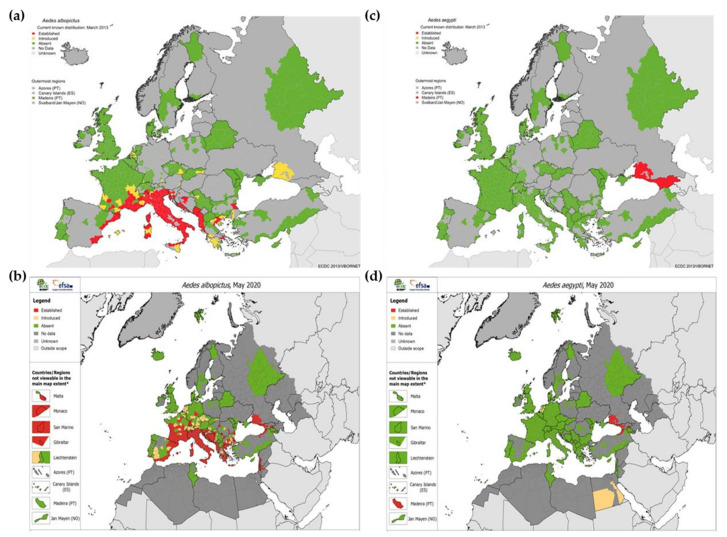
Expansion of *Aedes* spp. mosquitoes in Europe. Geographical distributions of *Aedes albopictus* in (**a**) March 2013 and (**b**) May 2020, and of *Aedes aegypti* in (**c**) March 2013 and (**d**) May 2020. Adapted from [219,220,221].

Regarding *A. aegypti*, it is possible that its first appearance in Europe occurred during the 17th century [31]. During the 1950s, it was reported in Spain and Portugal, being established in the Mediterranean countries, surrounding the Black Sea and the eastern region of the Caspian Sea [31,217,218]. After that, periodic recordings were found in Italy, Israel, and Turkey [31,217]. From 2004 on, the mosquito was reported in Madeira Island (Portugal) and in the northeastern Black Sea coast (Russia and Georgia) [31,217,218]. For both species, it is evident that between 2013 and 2020 they had been expanding, particularly *A. albopictus* (Figure 9).

Once the vector population becomes established, they are continuously available for transmission of the virus and if sufficiently abundant, so they are able to be effective carriers. The ECDC predicted that Portugal, eastern Turkey, Caspian Sea coast of Russia and the eastern Adriatic coast, Mediterranean basin, Greece, Turkey, and Balkan countries are European regions proposed as highly likely to become established with *A. albopictus* in Europe [195]. Indeed, as it can be seen in Figure 9B, *A. albopictus* is already established or becoming established in those suggested regions. Within the next decades, and taking into account the future possible climate changes, countries from Western Europe will afford the conditions for the establishment of *A. albopictus* [195,222]. Moreover, considering climate change trends, there will be an increased risk of expansion and establishment in northern Europe [195,222]. Importantly, *A. aegypti* is less likely than *A. albopictus* to survive and establish in temperate climate zones, limiting its expansion in Europe. Yet, coastal regions of the Black, Caspian, and Mediterranean seas might be appropriate for this mosquito [31,217]. With future climate changes and other factors, this trend may be altered [217].

Predictive studies of the expansion of *A. aegypti* and *A. albopictus* show the potential for a wider distribution of *A. aegypti* than *A. albopictus* [205], but only across tropical subtropical regions. In temperate regions of the planet, *A. albopictus* has a markedly broader distributional potential, now being found in Europe and USA (Figure 8) [205,223]. Although *A. aegypti* clearly requires warmer temperatures, a recent study [224] estimated that in each decade between 1950 and 2000, the world as a whole became 1.5% more suitable to the development of *A. aegypti*. It is predictable that this rate will increase from 1.5% to 3.2%−4.4% per decade by 2050 [224]. The rapid spread and establishment of both of these mosquito species in new regions constitutes a public health threat, as it increases the probability of new dengue and Zika outbreaks, highlighting the urgent need of successful treatments and/or prophylactic measures.

### 8.2. Mosquito Vectors Control

Regarding prevention of infectious diseases, as no successful treatments are available, vector control is an advantageous long-term strategy to manage them. For example, between 2000 and 2015, due to campaigns against malaria, *Plasmodium falciparum* infection prevalence was reduced by half and the incidence of the disease was reduced by 40% in endemic Africa, demonstrating that vector control campaigns are useful to control and reduce vector-borne diseases [225]. To maximize the effect, vector control measurements should target *Aedes* spp. mosquitoes in its immature and adult stages. The combined use of three approaches should be considered to improve its efficacy: environmental management, chemical control, and biological control [226]. Environmental management comprises environmental modification, environmental manipulation, and changes to human habitation/behavior, by destroying, altering, removing, or recycling non-essential containers providing habitat to any mosquito live stage. The maintenance and enhancement of urban infrastructures and basic services may help reducing *Aedes* spp. propagation [227]. They include improvement of water supply and water-storage structures, namely mosquito-proof water-storage containers, management of solid waste, street cleansing, and planning and construction of buildings (e.g., roof gutters should not be allowed) [227]. Regarding chemical control measurements, they comprise larvicide and adulticide (insecticides) [228]. However, insecticides might not be satisfactorily effective against *A. aegypti* and *A. albopictus* populations, due to the development of resistance [229]. Biological control, based on the use of organisms that will contribute to the reduction of the targets, including larvivorous fish and small crustaceans, have already proved to be effective [230]. Studies where *A. aegypti* is infected with specific strains of *Wolbachia* bacteria indicate that the transmission of flaviviruses may be reduced or even abolished [231,232,233].

Even though vector control may not be easy to implement throughout the community, there is some evidence of its success in controlling and reducing the presence of the vector and, consequently, the infectious disease they are responsible to transmit [3,234]. One of these examples was an eradication campaign of *A. aegypti* in South America, coordinated by the Pan American Sanitary Bureau during the 1950s and 1960s. In 1961, *A. aegypti* was not detected in 16 countries of the Western Hemisphere and yellow fever threat was almost abolished [235]. About a decade later, between 1980 and 1990, a vector control campaign was implemented in Cuba, right after the 1981 dengue epidemic, which was successful in controlling the outbreak [236]. In 1973, a control program in Singapore was implemented, leading to the reduction of *A. aegypti* population and low incidence of dengue for 15 years. However, since the 1990s, dengue incidence has increased [237]. These were successful strategies, but they were not sustained. In order to sustain and extend the improvements achieved over time, it is extremely important that the political will and investment are adequate to reach the desired goals [234,235]. In any case, a reduction of the number of infections and/or eradication programs, even if only of temporary success and requiring a renewal of efforts every year, are a worthy goal, given the number of lives and year adjusted life spans that may be gained. In addition, economic gains are more immediate and can positively feedback into a virtuous cycle of continued sustained vector and disease control public policy.

## 9. Concluding Remarks

There is a clear tendency of *Aedes* spp. mosquitoes to expand to new regions, consequently, setting the conditions for dengue and Zika diseases to spread. Urbanization, climate change, sociopolitical issues, and increasing travel and trade worldwide are factors that have a huge impact on that expansion. In order to control that negative effect, it is urgent to find strategies to be implemented across the community. A strong effort trying to discover effective treatments and prophylactic measures has been made. However, while these are not found, actions based on monitoring the virus vector, the infected people, and/or travelers from risk zones control are needed. For example, one measure implemented in some areas during the current SARS-CoV-2 pandemic that could be successfully extrapolated to dengue and Zika epidemics is the obligatory or recommended quarantine (when returning from regions where a disease outbreak is active). This could avoid the start of a cycle of autochthonous transmissions in the home country. This should be done only in countries where the mosquito populations are established. Moreover, as Zika can also be transmitted via sexual contact and mother-to-child, these modes of transmission should be also considered. In addition, up-to-date medical knowledge must be amply available and general practitioners must be made aware of the importance and mode of transmission of these vector-borne diseases. To accomplish that goal, political involvement, assistance, and support are critical. The relatively recent emergence of Zika virus and its quick spread across continents should have been a warning sign that other viral human pathogens would emerge and globally spread later on. As it is now common knowledge, viral epidemics can have devastating effects on countries’ economies and health systems. Since conditions are set (namely vector spread) for flaviviruses to emerge, they may cause major epidemic outbreaks, especially dengue and Zika viruses. Continued prevention efforts are highly effective measures, of major importance to avoid such outcomes, and, thus, flaviviruses prevention must become a key public health planning consideration in the future.

## Figures and Tables

**Table 1:** Number of reported and travel-associated cases of dengue and Zika in Europe [44].

Dengue			Zika	
Year	Reported Cases	Travel-Associated Cases	Reported Cases	Travel-Associated Cases
2015	2209	1960	29	29
2016	2823	2603	2119	2075
2017	2026	1918	275	264
2018	2191	2062	51	48
